# Investigation of *Escherichia coli* O157:H7 Survival and Interaction with Meal Components during Gastrointestinal Digestion

**DOI:** 10.3390/foods10102415

**Published:** 2021-10-12

**Authors:** Diane de La Pomelie, Sabine Leroy, Régine Talon, Philippe Ruiz, Philippe Gatellier, Véronique Santé-Lhoutellier

**Affiliations:** 1 Université Clermont Auvergne, INRAE, MEDIS, 63000 Clermont-Ferrand, France; dianedelapomelie@yahoo.fr (D.d.L.P.); sabine.leroy@inrae.fr (S.L.); regine.talon@inrae.fr (R.T.); philippe.ruiz@inrae.fr (P.R.); 2 INRAE, UR 370 QuaPA, 63122 Saint-Genès-Champanelle, France; philippe.gatellier@inrae.fr

**Keywords:** Shiga toxin-producing *Escherichia coli*, ground meat, gastrointestinal digestion, nitrite, nitroso compounds

## Abstract

*Escherichia coli* O157:H7 is responsible for foodborne poisoning, incriminating contaminated animal food and especially beef meat. This species can survive in the digestive tract, but, up to now, very few studies have considered its survival during the gastrointestinal digestion of meat. The present study aimed to investigate the survival of the pathogenic strain *E. coli* O157:H7 CM454 during the gastrointestinal digestion of ground beef meat and its interactions with meal components using a semidynamic digestive model. The CM454 strain in meat survived throughout digestion despite acidic pH (pH 2) and the presence of bile salts. The addition of nitrite and ascorbate in the digestion medium led to a decrease in strain survival. During digestion, a release of free iron was observed, which was accentuated in the presence of the CM454 strain. In addition, the strain modified the Fe^2+^/Fe^3+^ ratio, in favor of Fe^2+^ compared to the noninoculated meat sample. In the presence of nitrite, nitroso compounds such as nitrosamines, nitrosothiols, and nitrosylheme were formed. *E. coli* O157:H7 CM454 had no impact on N-nitrosation but seemed to decrease S-nitrosation and nitrosylation.

## 1. Introduction

Shiga toxin-producing *Escherichia coli* (STEC) are zoonotic agents that rank third among food-borne pathogens regarding their incidence and dangerousness in the European Union [1]. They are responsible for foodborne poisoning incriminating contaminated animal food products, vegetables, and watery drinks [1]. STEC, such as *E. coli* O157:H7, are considered to be a severe public health concern due to their low infection dose (10–100 CFU) and because of the seriousness of the syndromes caused, related, in particular, to their production of Shiga toxins [1,2,3]. Ruminants, especially beef cattle, are the primary reservoir of STEC and their feces are considered the main source of STEC contamination of carcasses in slaughterhouses and, therefore, of meat [4,5]. The consumption of bovine meat and products thereof accounted for 24% of epidemics caused by STEC in Europe in the period 2012–2017 [1]. According to an Ifop survey published in 2010, ground beef is considered the favorite meat product of children at 59% and can constitute a danger in the event of insufficient cooking (under 70 °C) of contaminated meat [6].

The survival of STEC after the ingestion of contaminated food throughout the human digestive tract depends on numerous factors and is not yet fully understood [7]. What is known is that *E. coli* O157:H7 can tolerate acidic pH values such as those found in the harsh stomachal environment (pH 1.5 to 3) [8]. Five acid resistance systems have been identified in STEC that allow them to resist a range of acidic pH [9,10]. The TIM (TNO gastrointestinal Model), which includes the stomach, duodenum, jejunum, and ileum, has been used to study the survival of *E. coli* O157:H7 in several homogenized and fluidized food matrixes such as ground meat, fermented olives, and cheese. The survival of *E. coli* O157:H7 was very low in the stomach and duodenum, while its transit through the distal parts (jejunum and ileum) resulted in an increase in the pathogenic population [11,12,13]. The survival could be impacted by the composition of the food matrix and its chemical transformation during digestion.

Indeed, the physicochemical environment of the stomach triggers chemical reactions, mainly oxidation, that can start during meat storage, namely the production of reactive oxygen species (ROS), leading to protein and lipid oxidation, further propagated during digestion [14,15,16,17]. The stomach environment enhances oxidation, due to its low pH and still dissolved oxygen content [18].

However, it should be considered that meat is part of a meal often accompanied by vegetables rich in vitamins and antioxidants. It has been established that vitamin C (or ascorbate) content can reach 1 mM in plant foods [19]. Moreover, when consuming vegetables, the significant input of nitrate should be taken into account, which is partly reduced into nitrite by endogenous buccal bacteria [20]. Therefore, vegetables containing around 400 mg/kg of nitrate will give approximately 60 mg/kg of equivalent nitrite or 1 mM in nitrite [21]. Under the acidic conditions of the stomach and in the presence of ascorbic acid (reduction medium), nitrite is reduced to NO (nitric oxide). Regarding chemical reactions, NO not only reacts with amino acids containing thiols and secondary amine groups, leading to the formation of nitrosothiols (S-nitrosation) and nitrosamines (N-nitrosation), respectively, but also with heme iron, leading to the formation of nitrosylheme (nitrosylation) [22]. Moreover, free iron present in meat can act as a powerful catalyst of oxidation and N-nitrosation during digestion [23].

Up to now, neither the survival during digestion of the pathogen *E. coli* O157:H7 in a complex meal nor its interaction with the macro and micronutrients present has been studied. The aim of this work was to investigate the survival of this pathogenic bacterium considering the chemistry of the meal components. To this end, a semidynamic digestive model representing the stomach and global intestinal compartments was set-up. The meal studied contained meat and nitrite/ascorbate content in a similar concentration to that occurring when eating vegetables.

## 2. Materials and Methods

### 2.1. Meat Samples

The reagents are listed in Supplementary Data S1. To avoid any animal effect, all the experiments were carried out on the two triceps brachii muscles from the same Charolais heifer. After 2 weeks of ageing under vacuum, the muscles were surface-sterilized using a Hot Air Gun (BOSCH^®^), then trimmed to 5 mm thickness. The meat was ground using 8 mm-hole mincers to mimic a bolus after chewing [24]. The meat bolus sample was used to perform protein and lipid assays according to the methods of Dumas [25] and Folch [26], respectively. The percentage of protein in meat was 20.8 ± 1.2%, while that of lipid was 0.2 ± 0.1%. The meat bolus samples were vacuum-packed in portions of 150 g and then frozen at −80 °C until use. For each experiment, the meat bolus was thawed at +4 °C overnight.

### 2.2. Strain Culture and Inoculation of Meat

The experiments were carried out using a nontoxigenic *E. coli* O157:H7 EDL933 Δ*stx* strain called CM454 that possesses a kanamycin resistance cassette [27]. The strain was cultivated in 3 × 100 mL of BHI medium (DIFCO™, France), in 100 mL Erlenmeyer flasks with screw caps, under low oxygenation and agitation (100 rpm) at 39 °C (temperature of cattle digestive tract, in which this bacterium is frequently found). The three flasks of culture were pooled when an optical density (OD) of 0.6 of the culture was reached. The culture was centrifuged (8000 rpm, 5 min, 4 °C) and the pellet rinsed and resuspended in 1.5 mL of physiological saline and kept on ice before inoculation in the meat bolus.

Each meat bolus sample of 150 g was inoculated with the 1.5 mL bacterial suspension to obtain an initial inoculation of 9 log CFU/g. The control meat bolus sample was prepared by adding 1.5 mL of sterile physiological saline. Each meat bolus sample—contaminated or not with the bacterial strain—was shaped to obtain a patty, which was placed on polystyrene trays, under an oxygen-permeable stretch film, and stored for 3 days at 4 °C.

### 2.3. In Vitro Batch Digestion Model of Meat Bolus and Sampling

The digestions (*n* = 3 per condition, control and EHEC, and time) were performed with an in vitro batch system, composed of five glass Erlenmeyer flasks of 100 mL (Supplementary Data S2). The first four flasks represented the stomach and the last one represented the intestinal compartment. The parameters (pH and enzyme content) were chosen to accurately reproduce human digestion from children to adults [28]. The temperature was kept constant at 37 °C. For each Erlenmeyer flask, there was a rotating system (100 rpm bar magnet), which mimicked stomach peristaltism and kept the bolus homogeneous. 

In the gastric compartment, 50 mL of simulated gastric fluid (SGF) was added (KCl 6.9 mM, NaCl 47.2 mM, CaCl_2_ 0.15 mM, KH_2_PO_4_ 0.9 mM, NaHCO_3_ 25 mM, MgCl_2_ 0.1 mM, and (NH_4_)_2_CO_3_ 0.5 mM) and the pH was adjusted to 2, according to the international consensus on digestion described by Minekus et al. (2014) [29]. After the addition of 10 g of meat, whose temperature had been raised previously to 12 ± 1 °C, the pH rose to 5 in a few minutes, due to the buffering capacity of the meat. The first sample, labeled t0/pH5, was obtained after the pH rose to 5, just before enzyme addition. For the other gastric samples, pepsin porcine gastric mucosa (Sigma-Aldrich, France) was added (2000 U/mL) and the gastric pH was lowered step by step by adding HCl 37% from pH 5 (initial pH of the meat in the gastric fluid) to pH 2 in 120 min. The second sample, labeled t40/pH4, was obtained forty minutes after the pH dropped to 4. The third sample, labeled t80/pH3, was obtained forty minutes after the pH dropped to 3; and the fourth sample, labeled t120/pH2, was obtained forty minutes after the pH dropped to 2.

In the intestinal compartment, the pH value was increased to 7 by adding 0.39% (*v*/*v*) of NaOH 10 N. Bile salts (50% cholic acid and 50% deoxycholic acid sodium salt mixture, Sigma-Aldrich, France) were added for a final concentration of 10 mM in the final mixture [29]. In addition, intestinal enzymes (Sigma-Aldrich, France) were added, namely trypsin (100 U/mL), chymotrypsin type II (25 U/mL), and intestinal lipase type II from a porcine pancreas (2000 U/mL). Digestion in the intestinal compartment lasted 120 min and the last sample was labeled t240/pH7.

In parallel, to reproduce a more complex meal, sodium nitrite (1 mM) and sodium ascorbate (1 mM), the concentrations found in vegetables, were added in flasks containing meat and after the pH rose to 5. Then, the same digestion protocol as that applied to the meat sample was used.

To sum up, two sets of gastro-intestinal digestion were carried out: meat contaminated or not with *E. coli* O157:H7 CM454; meat with nitrite/ascorbate contaminated or not with *E. coli* O157:H7 CM454. These sets of digestion were carried out in three independent replicates.

Four samples were taken: t0/pH5, t40/pH4, t80/pH3, and t120/pH2 for the gastric compartment. One sample was taken for the intestinal compartment: t240/pH7. At each digestion time, the Erlenmeyer flask contents were homogenized in stomacher bags with a filter (>250 µm) by mixing with a Stomacher double paddle blender (INTERSCIENCE BagMixer^®^ 400) for 1 min. The filtered digestates were collected and kept on ice for microbiological analyses or frozen at −80 °C for biochemical measurements.

### 2.4. Bacterial Enumeration

The initial microbiological quality of meat was controlled by enumerating the following: the total bacterial population on PCA medium (Difco™, Lyon, France) incubated at 30 °C for 72 h; lactic acid bacteria on MRS medium (Sigma-Aldrich, Illkirch, France) supplemented with nalidixic acid (40 mg/L) to inhibit Gram-negative bacteria and Delvocid (200 mg/L) to inhibit yeast and mold incubated at 25 °C for 72 h in a modified atmosphere jar (Anaerocult^®^ A, Lyon, Merck); *E. coli* on CHROMagar^TM^ O157 medium (Thermofisher, Saint Quentin Fallavier, France) incubated for 24 h at 37 °C; and kanamycin-resistant bacteria on BHI-kanamycin medium (50 µg/mL, Sigma-Aldrich, Illkirch, France) incubated for 24 h at 37 °C. To follow the survival of the CM454 strain during digestions, enumeration was carried out on BHI-kanamycin medium incubated for 24 h at 37 °C after appropriate serial dilutions.

### 2.5. Biochemical Analyses

#### 2.5.1. Protein Digestion Measurement

Protein digestion was determined by the fluorescamine method, which allows measurement of the free -NH_2_ groups from peptides and amino acids. The proteins were precipitated with cold trichloroacetic acid (15% final concentration) according to Sayd et al. (2016) to work only on released amino acids and peptides [30]. Fluorescamine (Sigma-Aldrich, Illkirch, France) is a fluorescent probe specific to the primary amine group (N-terminal α-amino group) of peptides and free amino acids [31]. The amine–fluorescamine complex, excited at 375 nm, emits at a wavelength of 475 nm. The standard is provided with a glycine solution. Thus, the level of peptides was expressed in millimolar (mM) equivalent glycine. The fluorescence measurements were performed with a Jasco FP-8300 spectrofluorometer. 

#### 2.5.2. Oxidation Measurement of Proteins and Lipids

The oxidation of the proteins from the digestate was evaluated by measuring the carbonyl groups, according to the method of Oliver et al. (1987) [32]. Carbonyl groups were detected by reaction with 2,4 dinitrophenylhydrazine (DNPH) (Sigma-Aldrich, Illkirch, France) which form protein hydrazones. The results were expressed as nanomoles of DNPH fixed per milligram of protein.

Lipid oxidation was measured by the thiobarbituric acid reactive substances (TBARS) method according to Lynch and Frei (1993) [33]. The results were expressed as nanomoles of equivalent malondialdehyde (MDA) per microliter.

#### 2.5.3. Determination of Free Iron Content and Its Oxidation State

Free nonheme iron from samples was transformed to heme iron by a centrifugation method using a Vivaspin^®^ 2 system (PES membrane with a cut-off of 5 kDa). Centrifugation (3800 g/h) was performed with a SL-40R centrifuge from Thermo Scientific. According to the Stolze method (1996) [34], the ratio of free iron content and Fe^2+^/Fe^3+^ forms was measured. Only the Fe^2+^ form could be linked with ferrozine reagent and formed a purple-colored complex with an absorbance at 540 nm. In the presence of reducing conditions with ascorbate addition, the total free iron was in Fe^2+^ form. Thus, we obtained the total iron content. Conversely, in the absence of ascorbate, the content in Fe^2+^ form was determined, and the content in Fe^3+^ form was obtained by subtraction. Absorbance measurements were performed in 96-well microplates with a Hitachi U-5100 spectrometer.

#### 2.5.4. Ascorbate Content and Its State of Oxidation

Total ascorbate content and the two forms of ascorbate (oxidized and reduced) were evaluated in the digestates by the method of Vislisel, Schafer, and Buettner (2007) [35]. In the presence of Tempol (4-hydroxy-2,2,6,6-tetramethylpiperidinyloxy) (Sigma-Aldrich, Illkirch, France), ascorbate was oxidized into dehydroascorbate (DHA), which then reacted with o-phenylenediamine (OPDA) (Sigma-Aldrich, Illkirch, France) to form the fluorescent OPDA–DHA complex. In the absence of Tempol, only the naturally oxidized ascorbate present in the medium could react with OPDA. The difference between the two measurements gave the level of reduced ascorbate. The fluorescence of the OPDA–DHA complex was measured in 96-well black polystyrene microplates with a Jasco FP-8300 spectrofluorometer (λ_ex_ = 375 nm and λ_em_ = 475 nm).

#### 2.5.5. Nitrite and Nitrate Content

Nitrite NO_2_^−^ and nitrate NO_3_^−^ contents were determined using the Griess reaction with a Sigma-Aldrich colorimetric assay kit (Sigma-Aldrich, Illkirch, France).

#### 2.5.6. Nitrosothiols and Nonvolatile Nitrosamine Contents

Nitrosothiols and nonvolatile nitrosamines were assessed according to the protocol of Bonifacie et al. (2021) [36]. Briefly, sample pH was increased to reach pH 8 with NaOH and to eliminate proteins and digestive enzymes, and the samples were centrifuged (3800 g/30 min) with a Vivaspin^®^ 2 system (PES membrane with a cut-off of 5 kDa). The assays were performed on filtrates. As mentioned in the previous paragraph, the initial contents of NO_2_^−^ and NO_3_^−^ were obtained on the filtrates using a Griess reaction colorimetric assay kit.

Nitrosothiol content was measured after saturating the samples with HgCl_2_ (40 mM), which specifically cleaves the S-NO bonds [36]. After filtration on 0.22 µm regenerated-cellulose (RC) membranes, nitrite content was measured and the subtraction between post-HgCl_2_-treatment and initial contents provided the nitrosothiol content.

Nitrosamine content was measured after irradiating the samples using a UV-lamp (UVItec Cambridge LF-215S Filtered UV Lamps, 254 nm Wavelenght, 2 × 15 W). It was established that the photodegradation of nitrosamines R-N = NO and nitrosothiols R-S = NO with UV irradiation leads to NO_2_^−^ and NO_3_^−^ release [36]. The samples were irradiated for 15, 30, 60, and 120 min. The NO_2_^−^ and NO_3_^−^ contents were measured at each irradiation time and the second-degree polynomial function curve f(1/t) = a * [NO_2_^−^ + NO_3_**^−^**]² + b * [NO_2_^−^ + NO_3_^−^]² + c was drawn, the c value giving the maximum content of nitrosothiols + nitrosamines. The subtraction between this value and the nitrosothiol content (obtained with the HgCl_2_ treatment) provided the nonvolatile nitrosamine content.

#### 2.5.7. Determination of Heminic Iron and Nitrosylation

Total heme iron content was determined in the form of acid hematin by extraction in acidic acetone [37] with a ratio of 80% acetone, 18% sample, and 2% HCl 12 N, and then filtrated on 0.22 µm RC membranes. The total heme iron content was evaluated by measuring the absorbance of the supernatant at 512 nm, with an absorption coefficient of 9.52 mM^−1^.cm^−1^.

The nitrosylheme iron was extracted in acetone with a ratio of 80% acetone and 20% sample and then filtrated on 0.22 µm RC membranes. The level of nitrosylheme in the filtrate was evaluated by measuring the specific absorbance at 540 nm with an absorption coefficient of 11.3 mM^−1^.cm^−1^. Absorbance measurements were performed on a Hitachi U-5100 spectrometer. Nitrosylation was expressed as the percentage of nitrosylheme to total heme iron.

### 2.6. Statistical Analysis

The values for each experimental condition are reported as the mean ± standard derivation (sd) of 3 independent determinations. To assess the effect of the different variables (time and pH of digestion, inoculation with bacterial strain, nitrite and ascorbate addition), the data were analyzed by ANOVA with a linear mixed-effects model with lme4 and lmerTest R packages [38] taking into account the interaction between the two variables, time and inoculum (fixed effects). Biological replicates are considered random factors. The Restricted Maximum Likelihood (REML) approach was used to estimate parameters. A Tukey’s post hoc test run at a 5% level of significance (*p* < 0.05) was performed after estimating the least-squares means with the emmeans R package [39]. A principal component analysis (PCA) was performed with the ade4 R package. All the statistical analyses were performed with R software.

## 3. Results

### 3.1. E. coli O157:H7 CM454 Survival and Its Interaction with Meat Components throughout Digestion

#### 3.1.1. *E. coli* O157:H7 CM454 Survived throughout Meat Digestion

The initial contamination of the meat samples after trimming, mincing, freezing, and thawing was low: 2.7 log CFU/g of total bacteria and 2.6 log CFU/g of lactic acid bacteria were enumerated. *E. coli*, *E. coli* serogroup O157, and kanamycin-resistant bacteria were not found.

The meat was inoculated at 9.8 ± 0.2 log CFU/g by the *E. coli* O157:H7 CM454 strain, and after storage for 3 days at 4 °C and 30 min at 20 °C to reach 12 °C, the enumeration was 8.6 ± 0.4 log CFU/g. Then, the population of the strain was determined at different sampling times from the gastric compartment to the ileum (Table 1). The strain was enumerated after several minutes at pH 5 in the gastric compartment, revealing 8.0 ± 0.5 log CFU/mL of gastric fluid. At the end of intestinal digestion, the bacterial population decreased to 6.7 ± 0.1 log CFU/mL of digestate. A percentage of 5% of the bacterial population was still alive at the end of digestion.

#### 3.1.2. *E. coli* O157:H7 CM454 had No Impact on Meat Protein Digestion, Contrary to Its Oxidation

In the gastric compartment, protein digestion kinetics assessed by free -NH_2_ release did not increase significantly (19.2 ± 1.5 mM eq glycine at t0/pH5 to 29.2 ± 3.6 at t120/pH2). At the end of intestinal digestion, peptide release reached 70.3 ± 5.7 mM eq glycine in the absence of *E. coli* O157:H7 CM454 and 62.3 ± 4.9 mM eq glycine in the presence of the strain. The final peptide content at t240/pH7 was similar for the two meat digestions, with and without *E. coli* O157:H7 CM454.

Protein oxidation was determined by measuring carbonyls. The carbonyl content was 33.4 ± 4.1 nmols/mg proteins at the beginning of digestion t0/pH5 and remained stable throughout meat digestion: 30.9 ± 4.6 nmol/mg protein at the end of gastric digestion t120/pH2 and 32.9 ± 5.2 nmol/mg protein at t240/pH7. During the digestion of the contaminated meat, the initial carbonyl content of 22.6 ± 5.0 nmol/mg protein remained stable (22.9 ± 0.6 nmol/mg protein) until the end of gastric digestion t120/pH2 and increased to 32.9 ± 2.3 nmol/mg proteins for t240/pH7. The factorial ANOVA analysis showed a significant effect of *E. coli* O157:H7 CM454 inoculation on the carbonyl content (*p* = 0.001).

**Figure 1:**
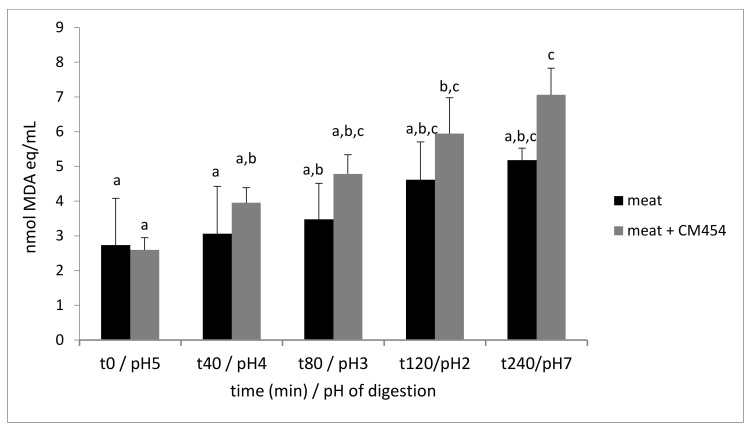
Lipid oxidation during digestion of meat inoculated or not with *E. coli* O157:H7 CM454. Values are mean +/− standard deviation of 3 independent biological replicates. Different letters indicate significantly different values (*p* < 0.05) by factorial ANOVA.

Lipid oxidation was determined by the measure of TBARS. The TBARS content in meat was 2.7 nmol of MDA eq/mL at the beginning of digestion and increased up to 5.2 nmol of MDA eq/mL at the end of digestion (Figure 1). In *E. coli* O157:H7 CM454-contaminated meat, lipid oxidation increased by 2.8-fold with a final content at 7.1 nmol of MDA eq/mL. The factorial ANOVA analysis showed a significant effect of *E. coli* O157:H7 CM454-inoculation on lipid oxidation (*p* = 0.005).

#### 3.1.3. *E. coli* O157:H7 CM454 did Not Impact Nitrosylation

**Figure 2:**
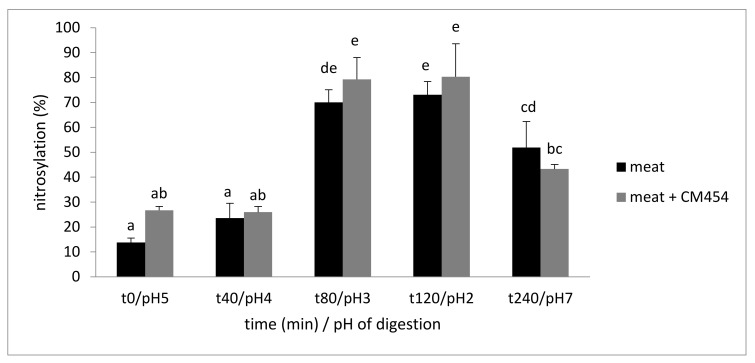
Evolution of the percentage of heminic iron nitrosylation during digestion of meat inoculated or not with *E. coli* O157:H7 CM454. Values are mean +/− standard deviation of 3 independent biological replicates. Different letters indicate significantly different values (*p* < 0.05).

The heme iron content was 46.1 ± 2.5 µM during meat digestion and 45.5 ± 3.0 µM during the digestion of contaminated meat. These values were similar and remained stable throughout digestion. The percentage of nitrosylation increased in the gastric compartment both in the noninoculated and inoculated meat (Figure 2). Maximal values were obtained at the end of gastric digestion, with 73.1% of nitrosylation for meat and 80.4% for meat with *E. coli* O157:H7 CM454. For these two digestions, a fall in the nitrosylation percentage was observed in the intestinal compartment, reaching 51.9% and 43.3% of nitrosylation for the noninoculated and inoculated meat, respectively. The factorial ANOVA analysis showed no effect of *E. coli* O157:H7 CM454 (*p* = 0.078) and only an effect of digestion (*p* < 0.001) on nitrosylation.

#### 3.1.4. *E. coli* O157:H7 CM454 Increased Free Iron Release and Modified Its Oxidation State

Free iron content was assessed throughout digestion (Table 2). The release of free iron was observed during the digestion of the noninoculated and inoculated meat. The time t80/pH3 seemed to be a pivotal point in our experiment. Free iron content had increased, reaching 6.6 ± 1.2 µM in the noninoculated meat and 7.5 ± 0.8 µM in the inoculated meat. Although this value remained stable until the end of digestion in the meat, it increased up to 15.3 ± 2.4 µM at the end of digestion, eightfold the initial rate in the presence of *E. coli* O157:H7 CM454. The factorial ANOVA analysis highlighted a significant effect of *E. coli* O157:H7 CM454 (*p* < 0.001), digestion (*p* < 0.001), and the interaction between these two factors (*p* < 0.001).

The Fe^2+^/Fe^3+^ molar ratios are shown in Table 2. During meat digestion, 89% of free iron was oxidized Fe^3+^ and 11% was reduced Fe^2+^, whereas during the digestion of contaminated meat, 47% of free iron was oxidized Fe^3+^ and 53% was reduced Fe^2+^. When the results were expressed in quantity, Fe^3+^ content was the same whatever the digestions of meat or contaminated meat up to the end of gastric digestion, but it was significantly different at the end of intestinal digestion (Table 2). *E. coli* O157:H7 CM454 inoculation led to an increase in free iron, with an Fe^2+^ content of 42%. The factorial ANOVA analysis highlighted a significant effect of *E. coli* O157:H7 CM454 (*p* < 0.001), digestion (*p* < 0.001) and the interaction between these two factors (*p* < 0.001).

### 3.2. *E. coli* O157:H7 CM454 Survival and Its Interaction with Meal Components (Meat, Nitrite, and Ascorbate) Throughout Digestion

#### 3.2.1. E. coli O157:H7 CM454 Survival Was Decreased by Nitrite/Ascorbate

The meat was inoculated at 9.3 ± 0.5 log CFU/g by the strain *E. coli* O157:H7 CM454, and after storage for 3 days at 4 °C and 30 min at 20 °C to reach 12 °C, the enumeration was 8.4 ± 0.6 log CFU/g. The strain was enumerated at t0/pH5 in the gastric compartment, revealing 8.6 ± 0.3 log CFU/mL gastric fluid (Table 3). The addition of nitrite and ascorbate in the meal resulted in a decrease of 3.5 log of the population throughout digestion, with a larger decrease (2.5 log) in the gastric compartment (Table 3).

In the presence of nitrite, 0.3% of the initial bacterial population was still alive at the end of gastric digestion and 0.04% at the end of intestinal digestion.

#### 3.2.2. *E. coli* O157:H7 CM454 Impacted Nitrite and Ascorbate Chemistry

Nitrite was added at a concentration of 1 mM and only 2/3 was recovered at t0/pH5. From the start of digestion, part of the nitrite (NO_2_) was oxidized into nitrate (NO_3_) in both the inoculated and noninoculated samples (Table 4). However, the ratio was 63% NO_2_/3% NO_3_ in the noninoculated meal and 81% NO_2_/19% NO_3_ in the inoculated meat. The nitrite rate decreased through time in the two digestions, up to time t80/pH3, and then remained stable. The factorial ANOVA analysis highlighted a significant effect of *E. coli* O157:H7 CM454 (*p* < 0.001), digestion (*p* < 0.001), and the interaction of these two factors (*p* = 0.011). The nitrate level decreased during the digestion in the meal sample (*p* = 0.036) but remained stable in the inoculated meal (*p* = 0.907).

Ascorbate 1 mM was added to the samples and recovered at the beginning of digestion (Table 4). The ascorbate content decreased similarly during the meal digestion with or without *E. coli* O157:H7 CM454. The factorial ANOVA analysis highlighted no effect of *E. coli* O157:H7 CM454 (*p* = 0.67), a significant effect of digestion (*p* < 0.001), and the interaction of these two factors (*p* = 0.026). Reduced/oxidized forms of ascorbate were also assessed. In the case of the meal digestion, 55% of the ascorbate was in the oxidized form dehydroascorbate (DHA) at the first time point t0/pH5, while at the third sampling time t80/pH3, a mean of 94% of ascorbate was oxidized up to the end of digestion. Conversely, during the digestion of the meal contaminated with *E. coli* O157:H7 CM454, 96% of the ascorbate was in the DHA form since the beginning of digestion and 4% was in the ascorbate form. The factorial ANOVA analysis highlighted a significant effect of *E. coli* O157:H7 CM454 (*p* < 0.001), digestion (*p* < 0.001), and the interaction of these factors (*p* < 0.001).

#### 3.2.3. *E. coli* O157:H7 CM454 Impacted Free Iron Release and Its Oxidation State

The free iron content was assessed throughout digestion (Table 4). It remained stable during the meal digestion, whereas the free iron content increased continuously during the digestion of the contaminated meal, from an initial value of 2.0 ± 0.4 µM to reach 12.6 ± 2.6 µM at the end of digestion, i.e., a sixfold increase. The factorial ANOVA analysis highlighted a significant effect of *E. coli* O157:H7 CM454 (*p* < 0.001), digestion (*p* < 0.001), and the interaction of these two factors (*p* = 0.002).

The Fe^2+^/Fe^3+^ forms were also determined (Table 4). At the beginning (t0/pH5) of digestion, approximately 20% of free iron was oxidized Fe^3+^ and 80% was reduced Fe^2+^. However, from the second time point t40/pH4, the ratio changed and 75% of free iron was oxidized Fe^3+^ in the meal, while the initial ratio remained unchanged throughout the digestion of the meal with *E. coli* O157:H7 CM454. The comparison of the meal digestions with or without *E. coli* O157:H7 CM454 highlighted an increase in Fe^2+^ release of 45% in the inoculated samples (*p* < 0.001).

#### 3.2.4. *E. coli* O157:H7 CM454 did Not Impact Protein Digestion or Lipid and Protein Oxidation

Similar initial peptide contents at t0/pH5 of 15.5 ± 1.3 and 15.9 ± 3.9 mM eq glycine were determined for both meal digestions, noninoculated or inoculated, respectively. No significant peptide release was noticed during gastric digestion in the absence of *E. coli* O157:H7 CM454. At t120/pH2, 15.2 ± 5.0 and 32.2 ± 9.2 mM eq glycine were determined in the absence and presence of *E. coli* O157:H7 CM454, respectively. However, at the end of intestinal digestion point t240/pH7, the peptide content was 2.9-fold higher in the noninoculated meal and 3.5-fold in the meal with *E. coli* O157:H7 CM454 (*p* = 0.05).

Protein and lipid oxidation did not change in the meal in the absence or presence of *E. coli* O157:H7 CM454. The content of carbonyls was 29.5 ± 4.3 nmol/mg proteins and 20.2 ± 6.1 nmol/mg proteins during the digestion of the meal and the inoculated meal, respectively. Similarly, lipid oxidation remained stable (<4.0 nmol of MDA eq/µL) throughout the meal digestion whether or not *E. coli* O157:H7 CM454 was present.

#### 3.2.5. *E. coli* O157:H7 Impacted Nitrosylation

Heme iron concentration was stable throughout both digestions: 52.5 ± 1.2 µM at the beginning and 50.9 ± 3.4 µM at the end of the meal digestion; and 57.1 ± 5.6 µM and 58.1 ± 4.2 µM in the presence of *E. coli* O157:H7 CM454. No effect of *E. coli* O157:H7 CM454 on digestion was shown.

**Figure 3:**
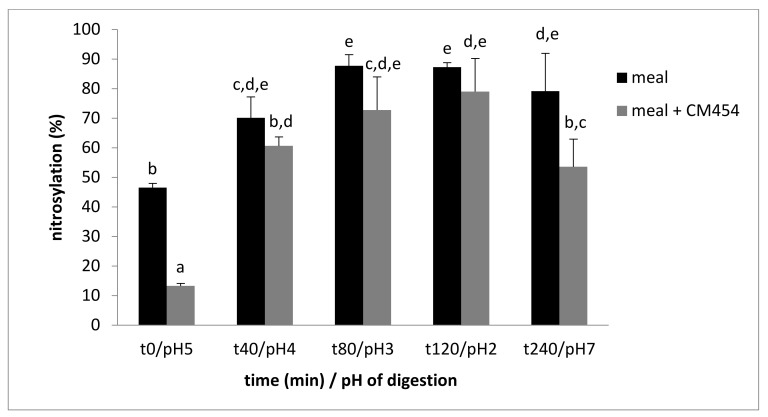
Evolution of the percentage of heminic iron nitrosylation during digestion of a meal (meat, nitrite, and ascorbate) noninoculated or inoculated with *E. coli* O157:H7 CM454. Values are mean +/− standard deviation of 3 independent biological replicates. Different letters indicate significantly different values (*p* < 0.05) by factorial ANOVA analysis.

However, the percentage of nitrosylation varied during digestion (Figure 3). It was 46.5% at the beginning of the digestion of the meal, it reached a maximum of 87.7% at t80/pH3, and it remained stable up to t120/pH2. In the contaminated meal, the percentage of nitrosylation began at 13.2% and rose to a maximum of 79.0% at point t120/pH2. Then, a significant decrease in iron nitrosylation of 25% was observed in this sample at the end of intestinal digestion.

The factorial ANOVA analysis showed a significant effect of *E. coli* O157:H7 CM454 (*p* < 0.001) and digestion (*p* < 0.001) on nitrosylation.

#### 3.2.6. *E. coli* O157:H7 CM454 did Not Impact N-Nitrosation but Impacted S-Nitrosation

**Figure 4:**
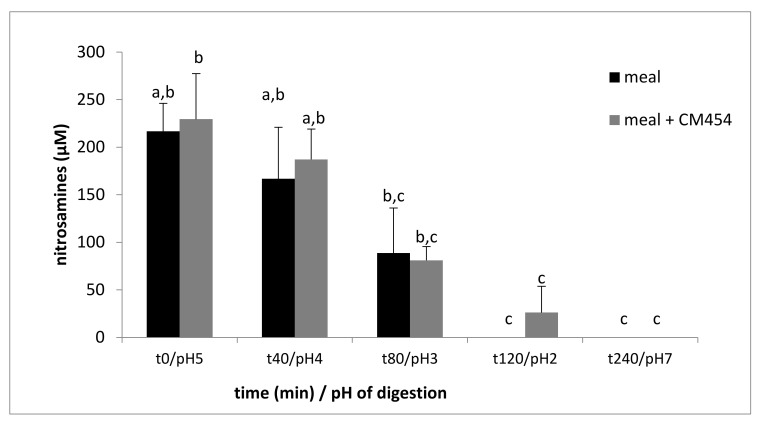
Evolution of nitrosamines content during digestion of a meal (meat, nitrite, and ascorbate) noninoculated or inoculated with *E. coli* O157:H7 CM454. Values are mean +/− standard deviation of 3 independent biological replicates. Different letters indicate significantly different values (*p* < 0.05) by factorial ANOVA.

A similar amount of nitrosamines (about 220 µM) was assessed from the beginning of digestion in both meal samples. Nitrosamine content decreased continuously throughout digestion until the end of gastric digestion (content divided by 8), and nitrosamines were no longer detectable in the intestinal compartment (Figure 4). The factorial ANOVA analysis showed a significant effect of digestion (*p* < 0.001) and no effect of *E. coli* O157:H7 CM454 (*p* = 0.391).

**Figure 5:**
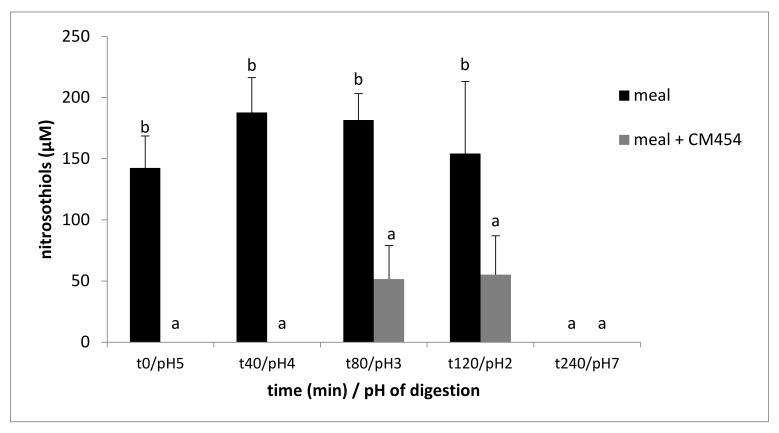
Evolution of nitrosothiols content during digestion of a meal (meat, nitrite, and ascorbate) noninoculated or inoculated with *E. coli* O157:H7 CM454. Values are mean +/− standard deviation of 3 independent biological replicates. Different letters indicate significantly different values (*p* < 0.05) by factorial ANOVA.

During the meal digestion, the nitrosothiol content was relatively stable during gastric digestion with a value fluctuating from 142.5 to 187.8 µM (Figure 5). Then, in the intestinal compartment, no nitrosothiol was detectable. Only traces of nitrosothiols were detected during the digestion of the contaminated meal. The factorial ANOVA analysis showed a significant effect of *E. coli* O157:H7 CM454 (*p* < 0.001), digestion (*p* < 0.001), and the interaction of these two factors (*p* < 0.001).

### 3.3. Principal Component Analysis (PCA)

**Figure 6:**
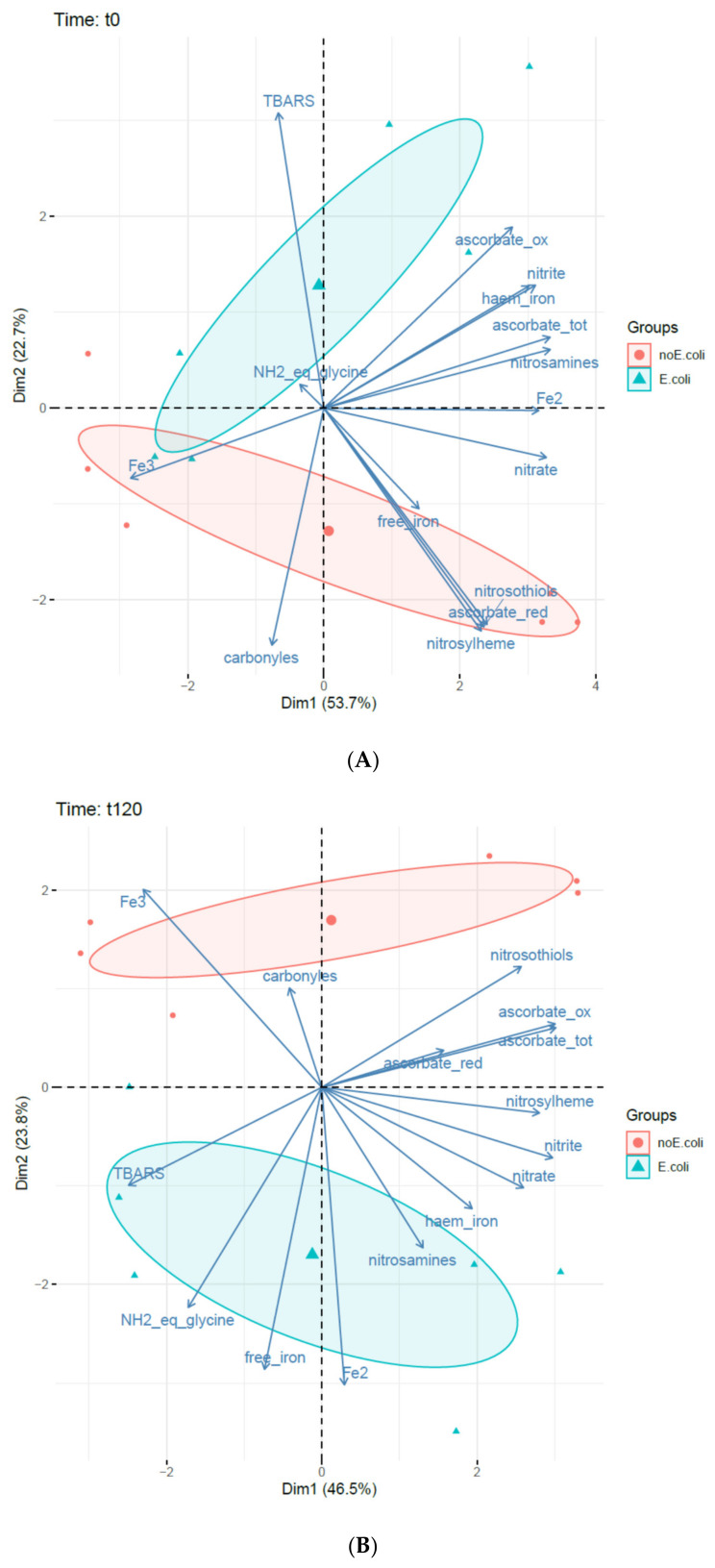

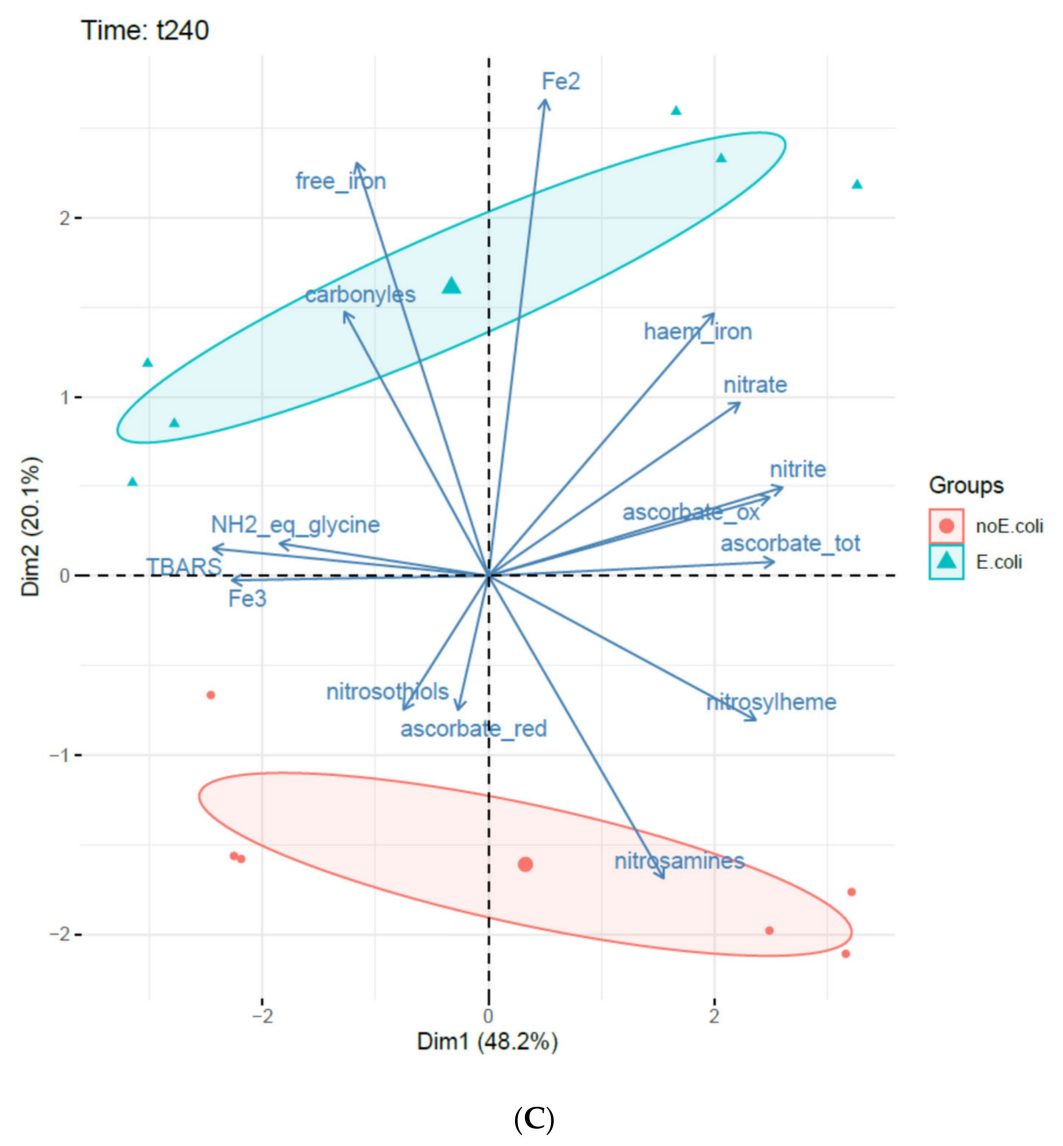
Effect of *E. coli* O157:H7 CM454 and nitrite/ascorbate on the biochemical measurements at different times of digestion. (**A**): PCA at t0/pH5; (**B**): PCA at t120/pH2; (**C**): PCA at t240/pH7.

PCAs were carried out on the complete dataset at t0, at the end of gastric digestion t120, and at the end of intestinal digestion t240 (Figure 6). These analyses illustrated the effect of *E. coli* O157:H7 CM454 on the biochemical evolution of meat during digestion.

At t0/pH5, 74.5% of the total variance was explained, with 53.2% for the first component and 21.3% for the second one (Figure 6A). The first axis corresponded roughly to the meal composition, and the two ellipses (*E coli*/no *E coli*) tended to split up.

At the end of gastric digestion, t120/pH2, 66.5% of the total variance was explained, with 44.2% for the first component and 22.3% for the second one (Figure 6B). At the end of intestinal digestion, t240/pH7, 68% of the total variance was explained, with 48.9% for the first component and 19.1% for the second one (Figure 6C). At 120 min, the first axis opposed nitrite/nitrate content to lipid oxidation of meat and the second axis corresponded to inoculation. Interestingly, the variables associated with inoculated meat were the amount of free iron and its reduced form Fe^2+^. At the end of digestion (t 240 min), a similar projection was observed, this time oxidized free iron correlated with lipid oxidation on the first axis, while E coli inoculation was still associated positively with free iron content.

## 4. Discussion

### 4.1. Static Digestive Model of Meat

To better understand the survival of *E. coli* O157:H7 CM454 during digestive conditions, the model used followed the static digestive system reported by Minekus [29] in the network of European researchers of Infogest. It is important to consider food buffering capacity during gastric digestion because it has an impact on intragastric pH and gastric secretion rate and possibly bacterial survival. Previous in vivo [40,41] and in vitro studies [42,43] reported a high buffer capacity of meat. The semidynamic model used mimicked meat buffering capacity as the pH of the gastric fluid increased from 2 to 5 after the addition of meat; then, HCl was added step by step to decrease the pH to 2 to mimic gastric secretion.

Similarly, the same concentrations of enzymes and digestive fluids and conditions described by Minekus were adopted [29]. These concentrations mimic the digestion of children (24 months) and adults [44] and thus were relevant for the purpose of studying the survival of *E. coli* O157:H7 responsible for severe symptoms, particularly in children.

### 4.2. Survival of E. coli O157:H7 during Digestion of Meat and Meal

In this study, the *E. coli* O157:H7 CM454 strain inoculated in ground meat could survive throughout digestion despite acidic pH. At the end of gastric digestion, t120/pH2, 5% of the initial bacterial population was still alive. This ability to resist an acidic environment could be attributed to the presence in *E. coli* O157:H7 of five acid resistance systems, with four dependent on amino acid decarboxylases (arginine, glutamate, lysine, ornithine, AR2, AR3, AR4, and AR5) and another called the glucose-suppressed system (AR1) [45,46]. The glutamate system is most effective at pH 2, while the arginine system is more efficient at pH 2.5 [46].

*E. coli* O157:H7 strain CM454 survived in the intestinal part when bile salts were added. Bile salts could act as an inhibitor via their detergent properties that enable the disruption of bacterial membranes [47,48,49] and via their action as DNA-damaging agents [20,49]. *E. coli* O157 has developed mechanisms of resistance to bile, including modified membrane structures that reduce bile permeability [7,48,49] and the active removal of bile acids due to efflux pumps [7,47,48,50,51].

The addition of nitrite and ascorbate, here mimicking the contribution of vegetables in the digestion medium, resulted in a drastic reduction in the *E. coli* O157:H7 CM454 population both at the end of gastric digestion (0.30% of the initial population) and of intestinal digestion (0.04% of the initial population). Compared to meat digestion, bacterial survival was reduced 5-fold in the gastric compartment and 51-fold in the intestinal one. The inhibitory property of nitrite could be attributed to the nitrosative stress generated [7,52]. The physicochemical conditions of the stomach such as oxygen pressure, low pH, and reducing conditions favor the formation of nitrite derivatives such as nitric oxide (NO). A synergetic effect of NO and acid pH has been observed on the reduction in survival of an *E. coli* O157 strain [53]. NO can react with DNA, proteins, and lipids, thereby resulting in antimicrobial activity [7,52]. *E. coli* O157:H7 possesses several resistance mechanisms against NO that could explain its survival. Up to now, four NO detoxification pathways have been characterized: flavohemoglobin HmpA [53], flavorubredoxin NorV [54,55], hybrid cluster protein Hcp–Hcr, and nitrite reductases NrfA and NirB [56,57].

### 4.3. E. coli O157:H7 CM454 Increased the Release of Iron

Free iron was released during meat and meal digestions. This release of free iron could be due to the acidification in the stomach and to the continuous proteolytic degradation of the iron-containing proteins, as has already been shown during the digestion of raw meat with or without nitrite [16]. The possible source of free iron could be either heme iron or storage proteins such as ferritin. Under our condition, the fact that the level of heme iron remained stable during digestion whatever the condition suggested a release of free iron from ferritin. Hoppler et al., (2007) reported the release of iron from pea ferritin under digestive conditions due to acid-induced structural alterations and the dissociation of proteins [56]. Interestingly, iron release was increased in the presence of *E. coli* O157:H7 CM454 in both digestions: 2.6-fold in meat and 4-fold in the meal at the end of the digestion. Iron is an essential micronutrient for bacteria, but it had to be closely regulated as over-accumulation results in an oxidative burden that could lead to cell death. To maintain iron homeostasis, *E. coli* has developed several mechanisms such as siderophores, citrate, and low-molecular-weight thiols such as ligands and iron storage proteins belonging to the ferritin family [57]. We can assume that *E. coli* O157:H7 CM454 grown in meat, an iron-rich medium, may have stored it and then released it during the lysis of many bacteria during digestion.

Finally, whether meat or meal digestion, the presence of *E. coli* O157:H7 CM454 modified the percentage of ferrous ion, which meant a more reducing medium, possibly due to oxygen consumption by the bacteria [58]. It could also be attributed to the role of ascorbate as an electron donor, as, in the presence of *E. coli* O157:H7 CM454, ascorbate was oxidized (DHA). 

### 4.4. E. coli O157:H7 CM454 Impacted the Formation of Nitroso Compounds

The heme iron was nitrosylated during digestion, with a different range according to digestion pH. Clearly, acidic pH enhanced the nitrosylation of myoglobin even in the absence of added nitrite. Meat contains residual nitrate possibly reduced to nitrite, as reported by Honickel (2008), possibly forming ●NO [59]. A recent study demonstrated the formation of nitrosylheme in a cooked ham model manufactured without sodium nitrite [60]. During digestion, increased myoglobin nitrosylation can be explained by the acidic and reducing conditions of the stomach that favors the formation of nitrous acid (HNO2) first, then nitric oxide (●NO). The reaction of nitric oxide with myoglobin differs according to the oxidation state of myoglobin. According to de La Pomélie et al. (2018) [16], the nitrosylation of metmyoglobin is reduced by 30% compared to oxymyoglobin. This reaction is also modulated by oxygen content in the medium. Interestingly, *E. coli* O157:H7 CM454 reduced myoglobin nitrosylation, particularly at the beginning and the end of the meal digestion. As ●NO is involved in nitrosylation, *E. coli* O157:H7 could trigger its detoxification systems, as mentioned above, and thus reduce nitrosylation.

During meal digestion, nitrosothiols were formed only in the gastric compartment. The nitrosating agent involved in nitrosothiol formation is also nitric oxide (NO●) [61]. The level of nitrosothiols was very low in the presence of *E. coli* O157:H7 CM454. This result could be related to the systems of detoxification of NO●, as already mentioned above. Moreover, this near-absence of nitrosothiols could be explained by the utilization of sulfur compounds such as cysteine or cysteine-containing peptide glutathione by *E. coli* [62]. It was reported that the decomposition rate of nitrosated glutathione is higher near physiological pH, in the presence of divalent ions such as Cu2+. These results could explain the nondetection of nitrosothiols in the intestinal compartment [63].

Nonvolatile nitrosamines were assayed at the beginning of the meal digestion, and then their amount decreased in the gastric compartment and no nitrosamines were detected at the end of digestion. This decrease has already been shown; it resulted in their degradation into several products not measured under our conditions [64]. In acidic medium, nitrous acid (HNO2) led to the formation of a nitrosonium cation (NO+), which is the nitrosating agent in N-nitrosation [65]. *E. coli* O157:H7 CM454 did not affect the formation of nitrosamines. This led us to assume that it did not interact with the nitrosonium cation. In a very different field, i.e., wastewater treatment, the fate of nitrosamines from sludge digestion was associated with their possible role as electron acceptors. In the case of NDMA (N-nitrosodimethylamine), it was shown nitrosamines can be used as an electron acceptor and reduced to ammonia and DMA (dimethylamine), in other words, the reduction of the nitroso group and subsequent N-N cleavage, explaining the loss of nitrosamines [66].

### 4.5. Oxidative Processes during Digestion

Lipid oxidation was recorded only during meat digestion. The addition of nitrite/ascorbate to the meat led to a stable level of oxidation whether or not in presence of bacterial contamination. In the products, the antioxidant effect of nitrite has been well documented in cured meat [65], and the action of nitrite against lipid oxidation during gastrointestinal digestion is based on the reaction of nitric oxide with superoxide radicals and lipid radicals to form stable LONO and unstable LOONO that decompose into stable LONO2 (NO● + L(O)O● → L(O)ONO), hence ending the oxidation process [23].

Contrary to the increase in lipid oxidation reported for meat digestion and enhanced by the presence of *E. coli* O157:H7 CM454, our results agree with those reported during the gastrointestinal digestion of beef meat carried out in vivo using cannulated pigs. The increase in lipid oxidation during beef digestion was lowered in the presence of antioxidant/polyphenols obtained from fruits and vegetables [66].

## 5. Conclusions

The *E. coli* O157:H7 CM454 strain inoculated in ground meat could survive throughout digestion, certainly due to its acid and bile resistance mechanisms. The addition of nitrite and ascorbate resulted in a drastic reduction in the *E. coli* O157:H7 CM454 population. Nitrite can lead to the formation of nitroso compounds such as NO responsible for nitrosative stress that affects the survival of the strain. However, *E. coli* O157:H7 possesses several NO detoxification mechanisms that could explain the reduction of nitrosothiols formed and myoglobin nitrosylation during the digestion of the meal contaminated by the strain. The formation of nitrosamines during digestion was not affected by *E. coli* O157:H7 CM454; this led us to suppose that it did not interact with the nitrosonium cation. Free iron was released during the digestions and this release was increased in the presence of *E. coli* O157:H7 CM454, possibly related to lysis of the strain. In order to better understand gene modulation in the digestive environment, transcriptomic analysis will be performed using RNA-sequencing.

## Figures and Tables

**Table 1:** Survival of *E. coli* O157:H7 CM454 along the meat digestion. Values are mean ± standard deviation of 3 independent biological replicates. Different letters indicate significantly different values (*p* < 0.05).

Time min/pH	t0/pH5	t40/pH4	t80/pH3	t120/pH2	t240/pH7
log CFU/mL	8.0 ± 0.5 ^a^	7.9 ± 0.4 ^a^	7.2 ± 0.5 ^a,b^	6.7 ± 0.8 ^a,b^	6.7 ± 0.1 ^b^

**Table 2:** Evolution of free iron content and Fe2+/Fe3+ forms during digestion of meat inoculated or not with *E. coli* O157:H7 CM454. Values are mean +/− standard deviation of 3 independent biological replicates. Different letters indicate significantly different values, at least *p* < 0.05, by factorial ANOVA.

	Absence of *E Coli*					Presence *E coli*					Time Effect	Inoculation Effect	Interaction
Time min /pH	t0/pH5	t40/pH4	t80/pH3	t120/pH2	t240/pH7	t0/pH5	t40/pH4	t80/pH3	t120/pH2	t240/pH7			
free iron µM	1.9 +/− 0.4 ^a^	2.0 +/− 0.4 ^a^	6.2 +/− 1.4 ^b^	6.9 +/− 1.4 ^b^	6.8 +/− 1.4 ^b^	2.0 +/− 0.4 ^a^	1.8 +/− 0.02 ^a^	7.4 +/− 0.7 ^b,c^	10.6 +/− 1.2 ^c^	15.3 +/− 2.3 ^d^	*p* < 0.001	*p* < 0.001	*p* < 0.001
Fe^2+^ µM	0.21 +/− 0.20 ^a^	0.36 +/− 0.23 ^a^	0.74 +/− 0.62 ^a^	0.66 +/− 0.62 ^a^	0.56 +/− 0.43 ^a^	0.97 +/− 0.22 ^a^	1.30 +/− 0.36 ^a^	3.55 +/− 0.67 ^b^	6.36 +/− 1.82 ^c^	5.64 +/− 0.09 ^c^	*p* < 0.001	p < 0.001	*p* < 0.001
Fe3^+^ µM	1.63 +/− 0.17 ^a^	1.67 +/− 0.30 ^a^	5.42 +/− 0.84 ^b^	6.20 +/− 0.84 ^b^	6.20 +/− 1.56 ^b^	1.02 +/− 0.41 ^a^	0.52 +/− 0.38 ^a^	3.89 +/− 0.24 ^b^	4.21 +/− 0.60 ^b^	9.69 +/− 2.40 ^c^	*p* < 0.001	NS	*p* < 0.001

**Table 3:** Survival of *E. coli* O157:H7 CM454 along the meal digestion in presence of nitrite/ascorbate. Values are mean ± standard deviation of 3 independent biological replicates. Different letters indicate significantly different values (*p* < 0.05).

Time min/pH	t0/pH5	t40/pH4	t80/pH3	t120/pH2	t240/pH7
log CFU/mL	8.6 ± 0.3 ^a^	7.7 ± 0.5 ^a,b^	6.4 ± 0.4 ^b^	6.0 ± 0.5 ^b,c^	5.0 ± 0.5 ^c^

**Table 4:** Evolution of nitrite, nitrate, total ascorbate, oxidized and reduced ascorbate, free iron content, and Fe^2+^/Fe^3+^ forms during digestion of meal inoculated or not with *E. coli* O157:H7 CM454. Values are mean ± standard deviation of 3 independent biological replicates. Different letters indicate significantly different values, at least *p* < 0.05, by factorial ANOVA.

	Absence of *E Coli*					Presence *E coli*					Time Effect	Inoculation Effect	Interaction
Time min/pH	t0/pH5	t40/pH4	t80/pH3	t120/pH2	t240/pH7	t0/pH5	t40/pH4	t80/pH3	t120/pH2	t240/pH7			
Nitrite	404 ± 28 ^a^	312 ± 70 ^a,b^	236 ± 43 ^b^	269 ± 71 ^a,b^	263 ± 68 ^a,b^	580 ± 58 ^c^	591 ± 64 ^c^	354 ± 35 ^a,b^	359 ± 40 ^a,b^	296 ± 47 ^a,b^	*p* < 0.001	*p* < 0.001	*p* < 0.001
Nitrate	241 ± 34 ^a^	258 ± 30 ^a^	142 ± 23 ^a,b^	96 ± 52 ^b^	82 ± 56 ^b^	139 ± 30 ^a,b^	179 ± 80 ^a,b^	182 ± 41 ^a,b^	187 ± 86 ^a,b^	145 ± 76 ^a,b^	*p* < 0.001	NS	*p* < 0.001
Total ascorbate	0.15 ± 0.06 ^a^	1.06 ± 0.06 ^a^	1.06 ± 0.03 ^a^	1.06 ± 0.05 ^a^	0.54 ± 0.21 ^c^	1.26 ± 0.12 ^a^	1.04 ± 0.08 ^a^	0.96 ± 0.15 ^a,b^	0.69 ± 0.17 ^b,c^	0.52 ± 0.02 ^c^	*p* < 0.001	NS	*p* < 0.001
Oxidized ascorbate	0.63 ± 0.08 ^a,b^	0.61 ± 0.07 ^a,b^	0.99 ± 0.18 ^b^	1.02 ± 0.10 ^b^	0.49 ± 0.22 ^c^	1.28 ± 0.08 ^b^	0.99 ± 0.12 ^b^	1.09 ± 0.29 ^b^	0.66 ± 0.14 ^c^	0.54 ± 0.12 ^c^	*p* < 0.001	*p* < 0.001	*p* < 0.001
Reduced ascorbate	0.51 ± 0.13 ^a^	0.44 ± 0.05 ^a^	0.08 ± 0.13 ^b^	0.06 ± 0.10 ^b^	0.04 ± 0.02 ^b^	0.006 ± 0.01 ^b^	0.05 ± 0.05 ^b^	0.006 ± 0.01 ^b^	0.033 ± 0.033 ^b^	0.034 ± 0.039 ^b^	*p* < 0.001	*p* < 0.001	*p* < 0.001
free iron µM	2.26 ± 0.36 ^a,b^	2.29 ± 0.24 ^a,b^	3.77 ± 0.44 ^ac^	4.39 ± 0.31 ^ac^	3.94 ± 0.40 ^ac^	2.01 ± 0.39 ^a^	6.70 ± 0.82 ^b,c^	7.67 ± 2.26 ^c^	11.28 ± 3.60 ^c,d^	12.56 ± 2.51 ^d,e^	*p* < 0.001	*p* < 0.001	*p* < 0.001
Fe^2+^ µM	1.79 ± 0.29 ^a^	0.66 ± 0.39 ^a^	1.73 ± 1.16 ^a,b^	0.71 ± 0.62 ^a^	0.35 ± 0.38 ^a^	2.12 ± 0.48 ^a,b^	6.06 ± 1.10 ^b^	5.71 ± 3.32 ^b^	9.38 ± 3.80 ^b,c^	10.02 ± 0.61 ^b,c^	*p* < 0.001	*p* < 0.001	*p* < 0.001
Fe^3+^ µM	0.48 ± 0.46 ^a^	1.63 ± 0.56 ^a,b^	2.04 ± 0.94 ^a,b^	3.67 ± 0.37 ^b^	3.59 ± 0.35 ^b^	0.38 ± 0.19 ^a^	0.58 ± 0.33 ^a^	1.55 ± 1.42 ^a,b^	1.96 ± 0.95 ^a,b^	2.09 ± 2.21 ^a,b^	*p* < 0.001	*p* < 0.001	NS

## Supplementary Materials

The following are available online at https://www.mdpi.com/article/10.3390/foods10102415/s1, Supplementary Data S1, Supplementary Data S2.
