# Supplementary material for: Investigation of Escherichia coli O157:H7 Survival and Interaction with Meal Components during Gastrointestinal Digestion
Source: Foods. 2021 Oct 12;10(10):2415. doi: 10.3390/foods10102415 (PMC8535473; doi:10.3390/foods10102415)

Supplementary Data S1: list of reagents

For microbial analyses, Plate Count Agar (PCA, DifcoTM, ref: 247940), Brain Heart Infusion Agar (BHI, DifcoTM, ref: 241830) and CHROMagar™ O157 (ref: 74002) were purchased from ThermoFisher France. The kanamycin antibiotic (ref: K1876), Man Rogosa Sharpe medium (MRS, ref: 69964) were purchased from Sigma-Aldrich France. For the digestions, the four enzymes used were purchased from Sigma-Aldrich France: pepsin from porcine gastric mucosa (ref: P6887), trypsin (ref: T0303), chymotrypsin type II (ref: C4129), intestinal lipase type II from porcine pancreas (ref: L3126) and bile salts (ref: 48305). For the biochemical analyses, fluorescamine probe (ref: P9015), 2,4-dinitrophenylhydrazine (DNPH, ref: D199303), thiobarbituric acid (TBA, reference), ferrozine reagent (ref: P5338), tempol (4-hydroxy-2,2,6,6-tetramethylpiperidinyloxy) (ref: 176141), o-phenylenediamine (OPDA, ref: P8287) and the Griess reaction colorimetric assay kit (ref: 23479-1KT-F) were all purchased from Sigma-Aldrich France.

Supplementary Data S2: workflow of the experimental design

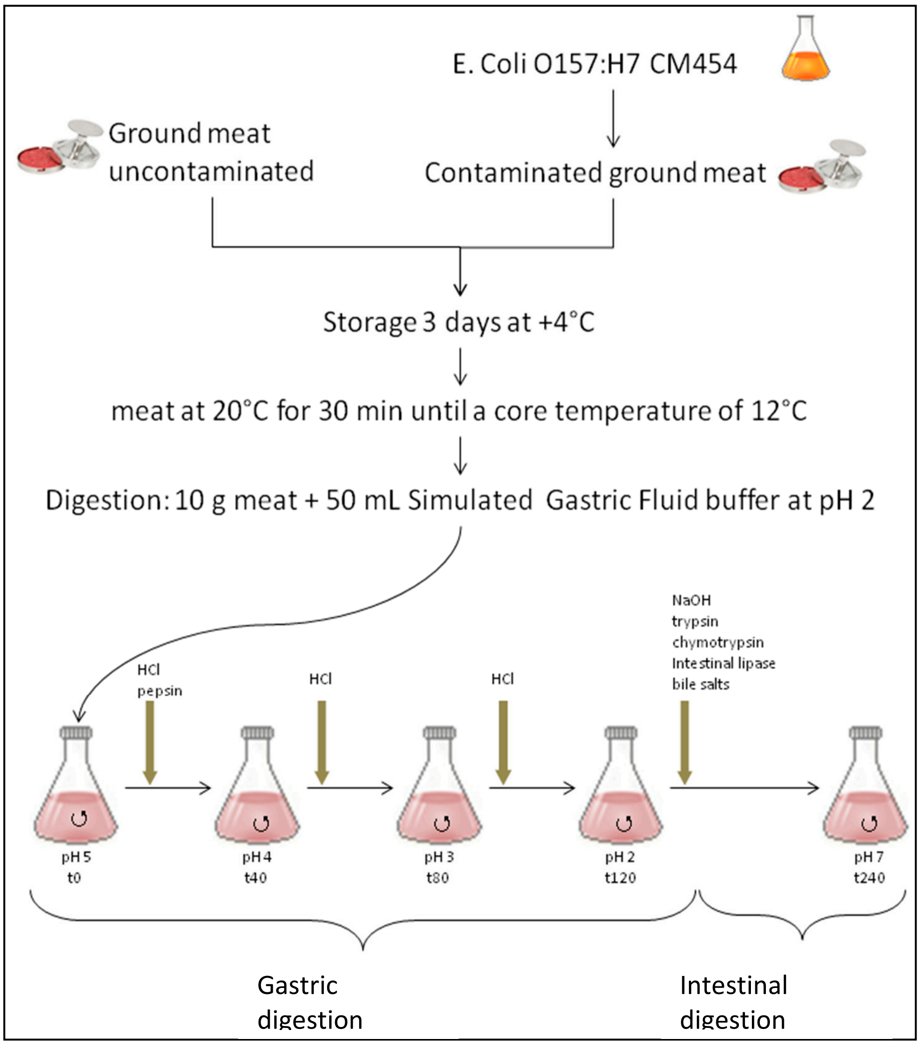

Supplement: Supplementary file 1 [file foods-10-02415-s001.zip › foods-1285007-supplementary.pdf]
